# Poor sensitivity of rapid tests for the detection of antibodies to the hepatitis B virus: implications for field studies

**DOI:** 10.1590/0074-02760160394

**Published:** 2017-01-30

**Authors:** Helena Medina Cruz, Leticia de Paula Scalioni, Vanessa Salete de Paula, Juliana Custódio Miguel, Kycia Maria Rodrigues do Ó, Flavio Augusto Pádua Milagres, Marcelo Santos Cruz, Francisco Inácio Bastos, Priscila Pollo Flores, Erotildes Leal, Ana Rita Coimbra Motta-Castro, Lia Laura Lewis-Ximenez, Elisabeth Lampe, Livia Melo Villar

**Affiliations:** 1 Fundação Oswaldo Cruz-Fiocruz, Instituto Oswaldo Cruz, Laboratório de Hepatites Virais, Rio de Janeiro, RJ, Brasil; 2 Fundação Oswaldo Cruz-Fiocruz, Instituto Oswaldo Cruz, Laboratório de Desenvolvimento Tecnológico em Virologia, Rio de Janeiro, RJ, Brasil; 3 Hospital São Lucas, Petrópolis, RJ, Brasil; 4 Universidade Federal do Tocantins, Faculdade de Medicina, Palmas, TO, Brasil; 5 Universidade Federal do Rio de Janeiro, Instituto de Psiquiatria, Rio de Janeiro, RJ, Brasil; 6 Fundação Oswaldo Cruz-Fiocruz, Instituto de Comunicação e Informação Científica e Tecnológica em Saúde, Rio de Janeiro, RJ, Brasil; 7 Universidade de Brasília, Brasília, DF, Brasil; 8 Universidade Federal do Rio de Janeiro, Campus Macaé, Macaé, RJ, Brasil; 9 Universidade Federal do Mato Grosso do Sul, Departamento de Bioquímica e Farmácia, Campo Grande, MS, Brasil

**Keywords:** hepatitis B virus, antibodies, rapid tests, performance

## Abstract

Rapid tests (RTs) can be used as an alternative method for the conventional diagnosis of hepatitis B virus (HBV). This study aims to evaluate antibodies to HBsAg (anti-HBs) and antibodies to HBeAg (anti-HBe) RTs under different Brazilian settings. The following three groups were included: GI: viral hepatitis outpatient services; GII: low resource areas; and GIII: crack users and beauticians. Imuno-rápido anti-HBsAg^™^ and Imuno-rápido anti-HBeAg^™^ RTs were evaluated and showed specificities greater than 95% in all groups. The sensitivity values to anti-HBs were 50.38%, 51.05% and 46.73% and the sensitivity values to anti-HBe were 76.99%, 10.34% and 11.76% in the GI, GII and GIII groups, respectively. The assays had a low sensitivity and high specificity, which indicated their use for screening in regions endemic for HBV.

Approximately 240 million individuals are chronically infected with hepatitis B virus (HBV) worldwide. Annually, 780,000 people die due to late-stage complications, such as cirrhosis and hepatocellular carcinoma (WHO 2015). In Brazil, between 2000 and 2012, 120,343 confirmed HBV cases were reported (BMH 2015). HBsAg prevalence is 0.63% in the north region, 0.31% in the midwest region and 0.31% in the southeast region (BMH 2015). Additionally, HBsAg prevalence among different groups (children, beauticians and crack users) is low (0% to 6.2%) in Rio de Janeiro (Santos-Cruz et al. 2013, Villar et al. 2014a, b).

During HBV infection, markers can be detected in sera samples using immunoassays. For example, patient sera that contain anti-HBs indicates immunity from past infections or vaccinations, and a patient with anti-HBe antibodies in their sera is considered as spontaneous resolution of infection or therapy-induced improvement (Gerlich 2013, Villar et al. 2015).

A diagnosis of HBV is done using enzyme immunoassays (EIAs) and electrochemiluminescence (ECLIA) (Gerlich 2013, Villar et al. 2015). However, the use of these assays in real-life conditions in low and middle-income countries has been challenging due to the relatively long period of execution, the necessity of well-trained personnel, good infrastructure and logistics (Soeung et al. 2009).

RTs are simple, easy, cheap and fast. Currently, several commercial RTs for HBsAg detection are available in Brazil, and BMoH has established policies and guidelines for HBsAg RT use (BMH 2016). The application of RTs as a substitute for conventional diagnoses of HBV infection may provide distinct advantages. Results are available in a few minutes, and RT use is feasible in poor infrastructure laboratories. HBsAg testing has shown high sensitivity, but some assays did not detect this marker, especially in low endemicity regions, such as Latin America. In these settings, it is recommended that additional HBV testing should be utilised for detecting other serological markers. Currently, few laboratories use RTs for other HBV markers, such as anti-HBs and anti-HBe. These assays could increase the identification of susceptible individuals for HBV vaccination and help in monitoring treatment response. Thus, in the present study, we evaluated the applicability of RTs for the detection of anti-HBs and anti-HBe markers in different groups to increase the availability of diagnosis in remote areas.

All individuals or parents/legal guardians signed the informed consent approved by the Ethics Committee of FIOCRUZ before recruitment. Serum samples were obtained from February 2009 to September 2013 from subjects who represented the following:

*Group I (GI)* - Consisted of 423 and 166 individuals who donated serum samples for the anti-HBs and anti-HBe RT evaluations, respectively. Individuals were referred from Viral Hepatitis Ambulatory (Oswaldo Cruz Foundation, FIOCRUZ). These individuals had suspected cases of viral hepatitis, and most of them lived in low socioeconomic areas in Rio de Janeiro state. Individuals were included if they were 18 years old or older, presented with either symptomatic or asymptomatic hepatitis, and had one or more risk factors for HBV infection.

*Group II (GII)* - Contained 1,526 and 788 individuals who donated serum samples for the anti-HBs and anti-HBe RT evaluations, respectively. These individuals lived in the following regions in Brazil: (1) Midwest Region - individuals from Pantanal of Mato Grosso do Sul state, a rural area 385 km from the capital of state, Campo Grande City; (2) North Region - subjects from rural communities in the city of Tocantinópolis in Tocantins state, 30 km from the urban area of the city; and (3) Southeast Region - employees from a private hospital located in Petrópolis and subjects living in an underprivileged community in Macaé (both cities in Rio de Janeiro). Individuals were included if they were not previously diagnosed as HBV infected.

*Group III (GIII)* - Consisted of 381 and 241 individuals who donated serum samples for the anti-HBs and anti-HBe RT evaluations, respectively. This group of individuals was considered to be highly vulnerable individuals (crack users in the southeast region of Brazil and beauty professionals in Rio de Janeiro state) likely to acquire or already have an HBV infection. Individuals included in this group were active crack users and beauticians, including manicurists, pedicurists, and hairdressers.

Sera samples were assayed for HBsAg, anti-HBc total, anti-HBc IgM and anti-HBs using commercial EIAs (Diasorin, Italy) and anti-HBe and HBeAg using commercial ECLIA (Elecsys anti-HBe and HBe, Roche Diagnostics, Mannheim, Germany) according to the manufacturer’s instructions. The OD/CO of positive/negative samples was defined according to the manufacturer for each test. All reactive samples were retested using the same assay in duplicate.

Both Imuno-rápido anti-HBsAg^™^ and Imuno-rápido anti-HBeAg^™^ RTs were evaluated (Wama, Minas Gerais, Brazil). According to the manufacturer’s instructions, the assays had 100% sensitivity and more than 98% specificity. These RTs were single-use, disposable-chamber, in vitro, qualitative, immunochromatographic assays that could detect anti-HBs and anti-HBe. The RTs allowed the detection of antibodies in serum, plasma and whole blood and used 100 µL of sample per assay. Each assay was completed in 10-15 min. Assays were conducted according to the manufacturer´s recommendations.

The sensitivity of the anti-HBs RT was also evaluated in the following two additional subgroups: (1) vaccinated individuals (isolated anti-HBs reactive) and previously HBV infected (anti-HBs/anti-HBc reactive) and (2) individuals with anti-HBs titers lower than 100 IU/mL and titers higher than 100 IU/mL. These cutoff values were chosen because approximately 85% of individuals who presented anti-HBs titers above 100 IU/mL after primary vaccination still had anti-HBs 22 years after vaccination.

The sensitivity of the anti-HBe RT was evaluated using the presence of an active HBV infection (anti-HBe reactive/HBsAg reactive), compared to a previous HBV infection (anti-HBe reactive/HBsAg non-reactive).

The detection of anti-HBs and anti-HBe in the serum samples by EIA or ECLIA was defined in the present study as the gold standard for the assessment of the sensitivity, speciﬁcity, positive predictive value (PPV), and negative predictive value (NPV) of each RT. To evaluate the deviation between the observed and expected values in each group, the X^2^ test or Fisher’s exact test were used. The Kappa coefficient (k) was used to assess the degree of agreement between the reference panel and the RT under analysis. Two-tailed *p*-values < 0.05 were considered statistically significant.

In this study, 2,330 individuals donated serum samples for the anti-HBs RT evaluation. The mean age ± standard deviation was 48 (± 13.99) years, 29 (± 20.39) years and 33 (± 14.41) years for GI, GII and GIII, respectively. Anti-HBs EIA for GI, GII and GIII showed 133, 572 and 107 reactive results, respectively, and 290, 954 and 274 non-reactive results, respectively. The sensitivity of the anti-HBs RT was relatively low in all groups, but a higher sensitivity was found in GII: 51.05% (292/572) compared to GI: 50.38% (67/133) and GIII: 46.73% (50/107). The specificity values were above 98.00% for all groups, and the concordance ranged from 53.32% to 57.66% in all groups (Table I).

**TABLE I:** Accuracy values (%) and frequency of true-positive, false-positive, true-negative and false-negative results for rapid tests for anti-HBs (Imuno-Rápido anti-HBsAg™) and anti-HBe (Imuno-Rápido anti-HBeAg™) among serum samples compared to gold standard (anti-HBs EIA: and anti-HBe ECLIA)

Parameters	Imuno-Rápido anti-HBsAg^™^				Imuno-Rápido anti-HBeAg^™^			
	
	Group I Reference centres (n = 423)	Group II Low prevalence (n = 1,526)	Group III High vulnerability (n = 381)	All groups (n = 2330)	Group I Reference centres (n = 166)	Group II Low prevalence (n = 788)	Group III High vulnerability (n = 241)	All groups (n = 1,195)
Sensitivity (CI%)	50.38% (41.63-59.10)	51.05% (46.92-55.16)	46.73% (37.05-56.59)	50.37% (46.94-53.80)	76.99% (68.18-84.39)	10.34% (48.43-18.76)	11.76% (14.57-36.41)	45.16% (38.43-51.97)
Specificity (CIs%)	99.66% (98.09-99.99)	98.01% (96.91-98.80)	98.54% (96.30-99.60)	98.42% (97.66-98.99)	96.23% (87.02-99.54)	100.00% (99.48-100.00)	100.00% (98.37-100.00)	99.80% (99.26-99.98)
PPV (CI%)	98.53% (92.09-99.96)	93.89% (90.62-96.27)	92.59% (82.13-97.95)	94.46% (91.86-96.41)	97.75% (92.12-99.73)	100.00% (66.39-100.00)	100.00% (15.81-100.00)	98.00% (92.96-99.76)
NPV (CI%)	81.41% (76.94-85.33)	76.95% (74.54-79.28)	82.57% (78.06-86.50)	78.76% (76.86-80.62)	66.23% (54.54-76.60)	89.99% (87.69-92.00)	93.72% (89.85-96.44)	89.13% (87.15-90.94)
K (CI%)	57.66% (48.36-66.96)	53.99% (49.31-58.67)	53.32% (42.58-64.06)	54.73% (50.85-58.61)	65.36% (53.66-77.06)	17.03% (0.00-34.51)	19.86% (0.00-59.13)	56.89% (49.61-64.17)
p-value	< 0.0001	< 0.0001	< 0.0001	< 0.0001	< 0.0001	< 0.0001	0.0047	< 0.0001
TP (n)	67	292	50	409	87	9	2	98
FN (n)	66	280	57	403	26	78	15	119
TN (n)	289	935	270	1494	51	701	224	976
FP (n)	1	19	4	24	2	0	0	2

CI: confidence interval; FN: false negative; FP: false positive; k: kappa; n: number of samples; NPV: negative predictive value; PPV: positive predictive value; TN: true negative; TP: true positive.

Samples that presented a false-negative result for anti-HBs in RT had low mean optical density/cut-off value ratio (OD/CO) values by ELISA compared to those samples considered anti-HBs true positive samples (Table II). Better sensitivity results of anti-HBs RT were observed in samples previously infected with HBV compared to vaccinated individuals in all groups and samples with anti-HBs titers higher than 100 IU/mL compared to titers lower than 100 IU/mL in all groups (Table III).

**TABLE II:** Mean values of OD/CO from EIE among false-negatives and true positive rapid tests for anti-HBs and anti-HBe detection

Group	Imuno-Rápido anti-HBsAg^™^			Imuno-Rápido anti-HBeAg^™^		
	
	Anti-HBs FN OD/CO mean ± SD	Anti-HBs TP OD/CO mean ± SD	p-value	Anti-HBe FN OD/CO mean ± SD	Anti-HBe TP OD/CO mean ± SD	p-value
Group I Reference centres	5.91 ± 5.44	29.56 ± 22.84	p < 0.0001	0.264 ± 0.316	0.025 ± 0.122	p < 0.0001
Group II Low prevalence	5.00 ± 6.46	35.21 ± 24.87	p < 0.0001	0.370 ± 0.357	0.099 ± 0.282	p = 0.0006
Group III High vulnerability	10.76 ± 8.98	30.60 ± 27.23	p < 0.0001	0.349 ± 0.422	0.005 ± 0.001	p = 0.1791
All groups	6.03 ± 7.02	33.67 ± 24.90	p < 0.0001	0.347 ± 0.357	0.032 ± 0.142	p < 0.001

FN: false negative; OD/CO: optical density/cutoff; SD: standard deviation; TP: true positive.

**TABLE III:** Sensitivity of the anti-HBs rapid test (Imuno-Rápido anti-HBsAg™) according to the anti-HBs titer and HBV status and sensitivity of anti-HBe rapid test (Imuno-Rápido anti-HBeAg™)

Evaluated groups	Anti-HBs				Anti-HBe	
	
	Sensitivity of anti-HBs rapid test according to HBV status		Sensitivity of anti-HBs rapid test according to anti-HBs titer		Sensitivity of anti-HBe rapid test according to HBV status	
		
	Previously, HBV infected^a^ n (%)	HBV vaccination^b^ n (%)	anti-HBs titers < 100 IU/mL n (%)	anti-HBs titers ≥ 100 IU/mL n (%)	Active HBV infection^c^ n (%)	Previously, HBV infection^d^ n (%)
Group I Reference centres	28/47 (59.57)	39/86 (45.35)	7/51 (13.72)	60/82 (73.17)	84/100 (84.00)	3/13 (23.10)
Group II Low prevalence	134/176 (76.14)	158/396 (39.90)	40/268 (14.92)	252/304 (82.89)	1/1 (100.00)	8/86 (9.30)
Group III High vulnerability	15/18 (83.33)	35/89 (39.32)	2/41 (4.87)	48/66 (72.72)	1/3 (33.30)	1/14 (7.10)
All groups	177/241(73.44)	232/571 (40.63)	49/360(13.61)	360/452 (79.65)	86/104 (80.77)	12/113 (10.62)

*a*: anti-HBs/anti-HBc reactive; *b*: anti-HBs alone; *c*: anti-HBe reactive /HBsAg reactive; *d*: anti-HBe reactive/HBsAg non-reactive; IU: international units; n: number of samples.

A total of 1,195 individuals were included in the anti-HBe RT evaluation. The mean age ± standard deviation was 45.3 (± 14.6) years, 31.6 (± 20.7) years, 30.4 (± 12.7) years for GI, GII and GIII, respectively. Anti-HBe results by ECLIA for GI, GII and GIII showed 113, 87 and 17 reactive results and 53, 701 and 224 non-reactive results, respectively. The sensitivities were 76.99% (87/111) in GI, 11.76% (2/17) in GIII and 10.34% (9/87) in GII. Specificity values were above 95.00% for all groups, and a high concordance between anti-HBe RT and EIA was observed in GI (k = 65.36%) (Table I).

False negative anti-HBe samples by anti-HBeAg RT presented higher mean OD/CO values by ECLIA compared to the true positive anti-HBe samples (Table II). The anti-HBe RT also had a higher sensitivity in those individuals who presented active HBV infection compared to those who were previously infected (Table III).

RTs are useful tools for HBV screening in low resource areas and/or emergency settings, but limited studies have been conducted to evaluate the performance of RTs for anti-HBe (El-Ghitany & Farghaly 2013) and anti-HBs (Oh et al. 1999, Whang & Um 2005, Cha et al. 2006, Bottero et al. 2013, 2016, 2016, El-Ghitany & Farghaly 2013, Wu et al. 2016). In the present study, two RTs (Imuno-Rápido anti-HBeAg^™^ and Imuno-Rápido anti-HBsAg^™^) were evaluated and showed high specificity and low sensitivities regardless of the endemicity profile of the populations analysed.

Anti-HBs RT showed high specificity values regardless of the endemicity profile of the populations evaluated and had similar values as those observed in France (97.80%), Taiwan (96.5%) and Egypt (95.08%) (Bottero et al. 2013, El-Ghitany & Farghaly 2013, Wu et al. 2016). These assays demonstrate an ability to identify true negative samples. Additionally, the sensitivity of anti-HBs RT was low in all groups (less than 51.5%), which is similar to what was observed using whole blood with Quick Profile (Lumiquick, USA) (58.08%) and sera samples with Advanced Test Quality One Step (Intec Products, China) (64.20%) (Bottero et al. 2013, El-Ghitany & Farghaly 2013). Furthermore, some studies have shown that the test sensitivity is above 91% in volunteers and children (Bottero et al. 2016, Wu et al. 2016), and a meta-analysis study showed a pooled sensitivity and specificity of 93.2% and 93.1%, respectively. However, RTs from different manufacturers were evaluated in those studies (General Biologicals Corporation, Genedia, Daewoong, Asan, Lumiquick diagnostics, and SD) (Shivkumar et al. 2012).

An interesting finding was the high sensitivity of the anti-HBs RT when samples with high values of OD/CO in EIE or high antibody titers (above 100 IU/mL) were used. The sensitivity of these anti-HBs RTs was the same as previously described (El-Ghitany & Farghaly 2013, Wu et al. 2016), showing that these tests may be useful for screening individuals with a high reactivity for anti-HBs. The RT for anti-HBs detection also demonstrated better performance in individuals with previous infections compared to individuals with a vaccine response. This difference may be due to the low anti-HBs titers obtained by vaccination in different circumstances, including immunosuppression, liver disease, renal failure, smoking, obesity, improper vaccine administration, short time interval between the second and third dose, age of the subject when vaccinated and the progressive decline of antibody titers in a large proportion of most individuals 9-11 years after vaccination (Kwon & Lee 2011).

In agreement with previous studies, anti-HBs RT and anti-HBe RT also demonstrated high specificity (above 96.00%) in all groups analysed, demonstrating an ability to discriminate negative results. The best performance of the anti-HBe RT was observed among patients referred to the Viral Hepatitis Clinic (65.36%) demonstrating the usefulness of this assay in the evaluation of seroconversion of HBeAg to anti-HBe in patients who have HBV. This is a favorable outcome that might be associated with a better prognosis among HBV patients according to the guidelines issued by the BMH (2011).

The anti-HBe RT was highly effective when samples with HBV active infection or samples with low values of OD/CO by ECLIA were considered. Conversely, El-Ghitany and Farghaly (2013) did not observe this finding which was probably due to differences in the RT and gold standard tests used.

In conclusion, RTs for anti-HBs and anti-HBe evaluated in the present study showed low sensitivity and high specificity indicating their use as a screening method in high HBV endemicity areas.
